# A citizen science approach to develop a digital intervention to reduce HIV stigma and promote HIV self‐testing among adolescents and young adults: a mixed methods analysis from Kazakhstan

**DOI:** 10.1002/jia2.26314

**Published:** 2024-07-19

**Authors:** Alissa Davis, Susan L. Rosenthal, Joseph D. Tucker, Olga Balabekova, Laura Nyblade, Yihang Sun, Denis Gryazev, Karsten Lunze, Sara E. Landers, Weiming Tang, Azamat Kuskulov, Valera Gulyayev, Assel Terlikbayeva, Sholpan Primbetova, Gaukhar Mergenova, the JasSpark Study Team

**Affiliations:** ^1^ School of Social Work Columbia University New York City New York USA; ^2^ Department of Pediatrics Columbia University Vagelos College of Physicians and Surgeons New York City New York USA; ^3^ Department of Psychiatry Columbia University Vagelos College of Physicians and Surgeons New York City New York USA; ^4^ Institute for Global Health and Infectious Diseases University of North Carolina‐Chapel Hill Chapel Hill North Carolina USA; ^5^ Clinical Research Department, Faculty of Infectious and Tropical Diseases London School of Hygiene & Tropical Medicine London UK; ^6^ Global Health Research Center of Central Asia Almaty Kazakhstan; ^7^ Research Triangle Institute Research Triangle Park Chapel Hill North Carolina USA; ^8^ Section of General Internal Medicine, Department of Medicine Boston Medical Center Boston Massachusetts USA; ^9^ Chobanian and Avedisian School of Medicine Boston University Boston Massachusetts USA

**Keywords:** adolescents, stigma, intervention, testing, low‐ and middle‐income countries, HIV

## Abstract

**Introduction:**

Kazakhstan has one of the fastest‐growing HIV epidemics in the world, with increasing rates among adolescents and young adults (AYA). Innovative strategies are needed to increase HIV testing uptake and decrease HIV stigma among AYA. Citizen science, defined as the active engagement of the general public in scientific research tasks, promotes and facilitates community engagement throughout the research process. This citizen science study used crowdsourcing to engage AYA in Kazakhstan to develop a digital intervention to reduce HIV stigma and promote HIV self‐testing. Our objectives in this paper are to describe the approach used, its feasibility and acceptability, and AYA motivations for and lessons learned collaborating on the study.

**Methods:**

From October 2021 to July 2022, in collaboration with a Community Collaborative Research Board and a Youth Advisory Board, we developed an open call requesting multimedia submissions to reduce HIV testing stigma. Eligible submissions were separated by age group (13−19 or 20−29 years) and judged by a panel composed of AYA (*n* = 23), healthcare professionals (*n* = 12), and representatives from the local government and non‐governmental organizations (*n* = 17). Each entry was reviewed by at least four judges and ranked on a 5‐point scale. The top 20 open call contestants were asked to submit self‐recordings sharing their motivation for and experience participating in the contest and lessons learned. Descriptive statistics were calculated for quantitative data. Qualitative data were coded using open coding.

**Results:**

We received 96 submissions from 77 youth across Kazakhstan. Roughly, three‐quarters (*n* = 75/96) of entries met judging eligibility criteria. Of the eligible entries, over half (*n* = 39/75) scored 3.5 or higher on a 5‐point scale (70.0%). The most frequent types of entries were video (*n* = 36/96, 37.5%), image (*n* = 28/96, 29.2%) and text (*n* = 24/96, 25.0%). AYA's primary motivations for collaborating on the study included a desire to improve society and help youth. The main challenges included creating content to address complex information using simple language, finding reliable information online and technological limitations.

**Conclusions:**

Crowdsourcing was feasible and highly acceptable among AYA in Kazakhstan. Citizen science approaches hold great promise for addressing the increasingly complex health and social challenges facing communities today.

## 1 INTRODUCTION

Eastern Europe and central Asia (EECA) has the world's fastest‐growing HIV epidemic with a 43.0% increase in incident cases of HIV acquisition from 2010 to 2020 [1] and for adolescents and young adults (AYA) rates are projected to increase 27.5% by 2030 [2]. Within EECA, Kazakhstan has the largest increase in incident cases of HIV [1], with a 132.7% increase in HIV incidence among AYA from 2018 to 2020 [3] coupled with low HIV testing rates (in 2015, 22.0% female; 15.0% male AYA tested) [4, 5]. Low uptake of HIV testing is due to a number of factors including perceived low risk of HIV acquisition, inconvenient testing locations and fear of HIV stigma [6, 7, 8, 9, 10]. Many in Kazakhstan are afraid to be tested due to concerns of severe discrimination if they test positive for HIV [7, 11, 12, 13]. In Kazakhstan, HIV testing is traditionally administered at city AIDS Centres, where it is obvious individuals are receiving HIV services. HIV self‐test kits recently became available in Kazakhstan, but are predominately targeted at men who have sex with men and the messaging may not resonate with AYA. Providing AYA with HIV self‐test kits allows them to access testing in a private location, thereby reducing the fear of involuntary disclosure of perceived HIV serostatus or assumed related sexual behaviours or substance use. AYA‐tailored messaging is needed to increase HIV testing in this group.

Innovative strategies are needed to generate tailored messaging and reduce HIV testing stigma among AYA in Kazakhstan. Citizen science is the active engagement of the general public in scientific research tasks [14]. Citizen science operates under a horizontal approach where community members are considered competent in‐the‐field experts [15]. It can engage vulnerable communities and promote health equity [16]. Not relying solely on public health experts fosters innovation and greater inclusion of perspectives from diverse community members, increasing ownership, relevance and sustainability of interventions [17].

Citizen science utilizes participatory methods, such as crowdsourcing, which engages a group of people to develop and share solutions to a problem [18]. Citizen science can be an effective way to develop community‐based solutions for a wide range of societal and health challenges, including HIV stigma [14]. Crowdsourcing often utilizes digital technologies, which have been shown to improve a variety of HIV‐related outcomes, including promoting HIV testing [19] and antiretroviral therapy adherence [20]. Digital technologies have also shown promise in reducing HIV stigma among healthcare providers [21] and internalized HIV stigma among people living with HIV (PLWH) [22].

The JasSpark Project (meaning “Young Spark” in the Kazakh language) is a citizen science study to engage AYA in Kazakhstan to develop a digital intervention to reduce HIV stigma and promote HIV self‐testing. The objectives of this paper are to describe (1) the citizen science approach used, (2) the feasibility and acceptability of using this approach to develop a digital intervention to reduce HIV stigma, and (3) AYA's perspectives on their motivations and learnings collaborating in the study.

## 2 METHODS

Our study used crowdsourcing to engage AYA in Kazakhstan to develop a digital HIV stigma reduction and HIV self‐testing intervention package. To address the first objective, we describe the process of implementation, including modifications that occurred during the study. To assess feasibility and acceptability, we describe Community Collaborative Research Board (CCRB) and Youth Research Collaborative (YRC) participation and the number and quality of open call submissions received. To assess AYA perspectives, we describe the findings from video recordings solicited from contestants with crowdsourcing entries ranked in the top 20. Descriptive statistics were calculated using SPSS (v28.0).

### 2.1 Description of approach used

**Figure 1:**
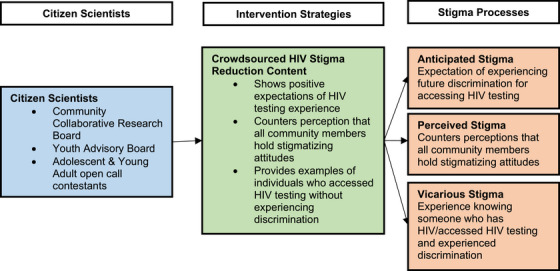
Citizen science study framework.

We launched an online crowdsourcing open call among AYA across Kazakhstan to develop intervention materials in Russian and Kazakh languages. The study was informed by the Theory of Planned Behavior, which posits that the intention to test for HIV is influenced by attitudes about HIV testing (including stigmatizing attitudes), perceived need, and an evaluation of the risks and benefits of testing [23, 24]. We used a Citizen Science Framework [25] integrated with stigma manifestations from the HIV Stigma Framework [26] to guide the study (see Figure 1).

#### 2.1.1 Establishment and meetings with the CCRB and YRC

AYA were involved in the JasSpark study through the team's YRC, which was comprised of two separate groups of youth. The first was recruited from non‐governmental organizations (NGOs) focusing on HIV among youth to partner on the study as part of the CCRB. These were AYA who were living with HIV and/or were engaged in youth activism in Kazakhstan (*n* = 8). As part of their role, with the other CCRB members, these AYA were responsible for engaging in more high‐level decision‐making, including the co‐development of study procedures, strategies for the open call, judging submissions and co‐development of a dissemination plan. Due to high interest from AYA not on our CCRB, we expanded participation to additional youth volunteers (*n* = 25). This second group consisted of AYA collaborators who heard about the study via word‐of‐mouth from CCRB members and Global Health Research Center of Central Asia (GHRCCA) staff and through announcements about the study at local universities, youth NGOs and on social media. The majority of volunteers in this second group were not living with HIV. AYA volunteers collaborated on a number of day‐to‐day study development aspects, including managing study social media accounts, co‐creating promotional materials for the study, providing feedback on the design of the submission portal and pilot testing it, and co‐designing and testing crowdsourcing procedures. Some AYA volunteers also helped judge crowdsourcing entries. AYA who helped judge entries received compensation for their time spent judging (27,000 tenge, ∼$60 USD). AYA were not financially compensated for involvement in other study activities. All AYA assisting with the study received a certificate of collaboration.

Our CCRB was comprised of the eight AYA mentioned above and representatives from youth local and international NGOs; Kazakhstan city, provincial and national AIDS Centres; youth health clinics; and media specialists working with youth. We had no strict selection criteria for the CCRB, but aimed to include a broad spectrum of professionals involved in working with youth. Given GHRCCA's long‐standing research presence in the region, research staff had many existing connections with NGO, health agency and other organization staff that work with youth. Many CCRB members had previous experience serving on CCRBs or collaborating on research, though the majority of AYA CCRB members did not have prior research collaboration experience. CCRB members were offered 27,000 tenge (∼$60 USD) compensation for their time and effort.

We worked with our CCRB to co‐create a solution that would allow for efficient collaboration and was mutually feasible and convenient for all. We had online meetings via Zoom and documentation was shared via email. We created a WhatsApp group based on feedback from AYA CCRB members to provide another outlet for sharing ongoing feedback and collectively discuss research process issues. To facilitate co‐creation with youth volunteers [15], we created a separate WhatsApp group and Telegram channel with the AYA volunteers and messaged them multiple times a week.

CCRB members and AYA volunteers received training on study procedures, defining stigma and judging processes. Attendance at meetings was tracked. Utilization of the diverse talents and strengths of our citizen collaborators greatly improved the development of study materials and the flow of study procedures. Citizen collaborators exhibited strong enthusiasm for the study, including significant in‐kind contributions of time and skills, requests by organizations to share crowdsourced materials on their websites, and positive feedback from contestants and volunteers with requests to become involved in other studies.

#### 2.1.2 Development of intervention materials: crowdsourcing open call

**Figure 2:**
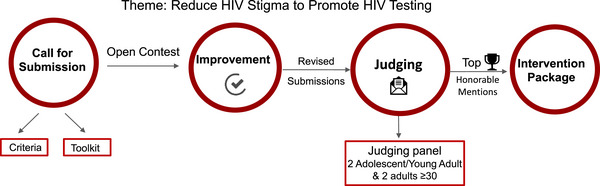
Develop a crowdsourced intervention package.

Our collaborative process (Figure 2) began with posting an open call on our study website [27] and various social media channels (e.g. Instagram, Tiktok, Facebook, WhatsApp, Telegram) and through youth events (in‐person and online). We developed a national crowdsourcing open call, inviting AYA ages 13−29 years living in Kazakhstan to submit multimedia entries. YRC members created a video to promote the contest and managed study social media accounts. CCRB and YRC members provided feedback on the study website and participated in livestream events to promote and provide more information about the contest. The call focused on developing submissions to reduce HIV stigma to promote HIV self‐testing among at‐risk AYA in Kazakhstan. It contained a toolkit with basic information about stigma and HIV, as well as a list of free software and resources to aid in the development of materials. Due to the presence of stigmatizing content in early submissions, we added additional information about avoiding stigmatizing language based on guidelines from HIV‐focused organizations (e.g. UNAIDS, UNICEF) to the open call instructions.

To be eligible to submit, AYA had to be 13−29 years old and live in Kazakhstan. They were allowed to submit multiple submissions, either individually or as a group. Eligible submission formats included video, audio, text, images/photos and other multimedia content (e.g. online games, webpages, crossword puzzles). Submissions could be in Russian or Kazakh. Prior to submission, all contestants had to complete a Multimedia Release Form (also signed by a parent for AYA under age 18) and Contestant Agreement providing their permission to use their content as part of a research study and in presentations and agreeing they would postpone publishing their materials until after completion of the scientific study. AYA in the YRC were eligible to submit entries to the crowdsourcing open call, but none submitted.

All submissions were screened for eligibility and the presence of stigmatizing content prior to judging. Each entry was first reviewed by two GHRCCA research staff (OB, DG), and then all entries were reviewed by the Kazakhstan‐based PI (GM). Contestants who had entries with stigmatizing content were provided feedback by research staff via their preferred communication method (e.g. WhatsApp, email) and given a chance to revise and resubmit entries. Those who chose to resubmit had only their revised entry judged, while those who chose not to resubmit had their original entry judged.

Eligible submissions were divided by age group (13−19 years old or 20−29 years old) for evaluation by a judging panel consisting of Kazakhstani AYA (*n* = 23), healthcare professionals (*n* = 12), and representatives from the local government and NGOs (*n* = 17). Each entry was judged by two AYA in our YRC (volunteers and AYA CCRB members) and by two other CCRB community partners (i.e. AIDS Centre, youth clinic or NGO staff). To obtain diverse perspectives while easing judging burden, each judge rated a maximum of five entries, thus all entries were not rated by the same four judges. We distributed entries across judges to ensure each judge received a comparable mix of different content types (e.g. video, image). Entries were ranked on a 5‐point scale based on four judging criteria used in previous crowdsourcing studies [28, 29]: (1) potential to reduce HIV stigma to increase HIV testing; (2) innovation; (3) relevancy to youth; and (4) overall impression. Entries were considered high‐quality if they scored an average of 3.5 or higher on a 5‐point scale between the four judging scores. All contestants received participation certificates. A virtual awards ceremony was conducted to honour awardees. First place was awarded for the top Russian and Kazakh language entries in each age category (13−19 years and 20−29 years) and received 220,000 tenge (∼$485 USD). Second‐place entries in each age category received 132,000 tenge (∼$290 USD) and third‐place entries received 67,000 tenge (∼$145 USD). Seventeen contestants received honourable mentions and received 27,000 tenge (∼$60 USD). Multimedia content from winning submissions were combined to form the intervention package that would be tested in a subsequent randomized control trial. Final intervention materials were adjusted for clarity and to correct errors.

To explore the motivations and learnings of AYA collaborators, we messaged the top 20 open call contestants (determined via the 10 highest average judging scores in each age category) and asked them to submit self‐recordings responding to the prompts: (1) Why did you decide to take part in this competition? (2) What new things did you learn while working on your content? (3) What was the hardest thing about creating content? Due to resource limitations, we were not able to gather feedback from all 77 contestants. The top 20 entries included entries in both Russian and Kazakh and across media types (e.g. video, image, text). Submission of self‐recordings was optional. Interested contestants (*n* = 13) sent self‐recorded videos via a messaging app to GHRCCA research staff. An initial coding structure was developed based on the prompts sent to the contestants, and then refined through an iterative review process by the research team. The coding of each recording was conducted by at least three members of the research team. The data collection process (from initial meetings with the CCRB to the sharing of self‐recordings) was conducted between October 2021 and July 2022. All study procedures were reviewed and approved by Columbia University's Institutional Review Board and Al‐Farabi Kazakh National University's Ethics Committee.

## 3 RESULTS

### 3.1 Assessing feasibility and acceptability

#### 3.1.1 CCRB and YRC feasibility and acceptability

The CCRB (*n* = 25, including 8 AYA; 20.0% male, 80.0% female, age range 14−73) met twice before launching the open call to determine content and procedures. Attendance was high—96.0% (*n* = 24) during the first meeting and 80.0% (*n* = 20) during the second meeting. We held a third meeting with the CCRB to review judging procedures (attendance 80.0%, *n* = 20). Seven of the eight AYA CCRB members participated in the judging process. CCRB members were invited to attend the virtual awards ceremony following the judging process (52.0% attended, *n* = 13). CCRB members spent an average of 8−10 hours contributing to the study. Seven of the eight AYA CCRB members also served as AYA volunteers. AYA volunteers (*n* = 25, 60.0% male, 40.0% female, age range 14−31) were highly active in collaborating on the study; of the 25 volunteers, 23 (92.0%) helped conduct at least one component of the study (e.g. develop promotional materials, disseminate study information via social media, participate in judging). Among the AYA volunteers who were not CCRB members, six served as judges. The time AYA volunteers spent collaborating on the study ranged widely. On the low end, some partners spent a few hours total on all activities, while on the high end, partners spent several hours each week over the duration of the study period.

#### 3.1.2 Open call feasibility and acceptability

During the 4‐month open call period, 3412 individuals visited the website. We received 96 submissions from 77 youth (28.6% male, 71.4% female) across Kazakhstan. Eleven youth submitted two entries and four submitted three entries. Nearly, two‐thirds (64.6%, *n* = 62/96) of entries were from contestants between ages 13 and 19.

Roughly, three‐quarters (*n* = 75/96) of entries met judging eligibility criteria. Entries were excluded if they were unrelated to HIV testing or stigma reduction, were low quality, plagiarized and/or had highly stigmatizing content. The average score for all entries was 3.4 on a 5‐point scale. Of the eligible entries, over half (*n* = 39/75) scored 3.5 (70.0%) or higher. Inter‐rater agreement between the judges was low (Fleiss’ kappa = 0.05, *p* = 0.04). The most frequent types of entries were video (*n* = 36/96, 37.5%), image (*n* = 28/96, 29.2%) and text (*n* = 24/96, 25.0%), with a few audio (*n* = 3/96, 3.1%) and other (*n* = 5/96, 5.2%) entries. Thirty contestants had stigmatizing content or misinformation in their submissions. Stigmatizing content included stigmatizing language (e.g. HIV‐infected), stigmatizing images (e.g. blood and skulls), and misinformation and exaggerated fears around HIV transmission (e.g. high risk of HIV acquisition in nail salons). Of the 30 contestants who received feedback on stigmatizing content, 10 revised and resubmitted their entries, and 80.0% of resubmissions (*n* = 8/10) no longer contained stigmatizing information.

### 3.2 AYA collaborator motivations and learning

Thirteen out of 20 top contestants sent self‐recordings. AYA described their motivations for participating, lessons learned from participation and challenges creating content.

#### 3.2.1 Motivations for participation

Seven contestants expressed their desire to improve society or help others feel supported as a key motivation for participating in the contest. Additionally, six AYA contestants were artistic and expressed wanting to develop creative materials or use their skills.

#### 3.2.2 Lessons learned from participation

All contestants reported learning something new about HIV, stigma and/or testing. A number of contestants reported learning that PLWH can live long and normal lives. Several contestants also mentioned learning about the ability for PLWH to give birth to children without HIV, indicating a persistent misperception in Kazakhstani society. Contestants also reported learning more about the challenges faced by PLWH, including children with HIV. Some contestants also reported using the knowledge and skills they gained from participating to design crowdsourcing projects to address other societal problems.

#### 3.2.3 Challenges creating content

Many contestants discussed the difficulty in creating content that could convey complex information using simple, non‐stigmatizing language. Contestants wanted their work to have a positive impact and struggled to develop compelling messaging. AYA also discussed the difficulty in sifting through stigmatizing information online to find reliable sources. Many AYA reported not being aware of HIV stigma themselves and needing to search for reliable information to become more informed. For some AYA, this included meeting with HIV specialists or other professionals. Contestants also reported some technological challenges in creating content. Although some AYA had extensive previous experience with video editing, audio and graphic software, other AYA had limited exposure to these types of tools and had to learn how to use them.

## 4 DISCUSSION

The JasSpark Study used a citizen science approach to develop digital intervention materials to reduce HIV stigma and promote HIV self‐testing among AYA. There is limited research on citizen science approaches to stigma reduction [28]. Most research aimed at HIV stigma reduction has used education‐based, skills‐building and/or counselling approaches with public health experts [6, 30, 31]. However, citizen science approaches have been used to address other issues facing AYA, such as school community wellbeing [32], barriers to physical activity [33], nutrition [34] and asthma [35].

The study serves as a useful model for designing inclusive methods to broaden public engagement in addressing stigma. Compared with other studies using crowdsourcing among diverse populations, we received a large number of submissions and a high percentage of high‐quality submissions [18, 36], indicating high acceptability. Our findings indicate that crowdsourcing is a feasible citizen science approach to use among AYA in central Asia. A challenge in citizen science projects is finding engaged volunteers. While some projects have hundreds of volunteers, in some studies, less than 10.0% actively make contributions [37]. However, active participation among our AYA volunteers was high—greater than 90.0%, indicating citizen science approaches may be particularly well‐suited for engaging AYA, particularly on topics they consider important. Of note, our AYA volunteers received no monetary compensation, only certificates of collaboration. Given that many AYA are applying for colleges or jobs and such certificates are valuable for their resumes, this may have been a motivating factor for their collaboration.

Motivations for participating in citizen science projects can vary, but often include reasons related to values (e.g. humanitarian concerns for others), understanding (e.g. opportunity for learning new skills/knowledge), social (e.g. opportunity for interacting with others), career (e.g. obtain career‐related benefits) and protective (e.g. reduce guilt over being more fortunate than others) [37]. The majority of AYA citizen scientists in our study cited pro‐social motivations. Many AYA had a strong desire to improve society and help youth or use their creative talents for good. Crowdsourcing provides AYA an opportunity to use their creative skills in a competitive forum, which may provide a way to engage them in an important topic they might not otherwise engage in.

Our study also highlighted the challenges associated with addressing stigma via citizen science approaches. Some crowdsourced materials developed by citizens could increase stigma, consistent with other literature [14]. Nearly, a third of submissions contained stigmatizing content, indicating a need for vetting and refinement of community contributions. Approximately one‐third of contestants revised submissions based on feedback, demonstrating a desire to learn. Open call submissions also served as a useful window to highlight where some sources of societal HIV stigma were stemming from—in this case, primarily around misperceptions and exaggerated fears about how HIV is transmitted. This is valuable for the design of future research studies and programmes to address HIV stigma in Kazakhstan and central Asia.

This study illustrates the promise of using a citizen science approach to develop HIV stigma reduction interventions. Strengths of this approach included strong participation from citizen scientists, including AYA; a large proportion of high‐quality submissions; and the development of highly creative and innovative intervention content. We also implemented strong quality control procedures; all submissions in our study were reviewed by at least three people to determine whether the material included stigmatizing content.

However, there are some limitations. First, because this study was implemented in a real‐world environment, we were not able to fully control all study processes (e.g. number of submissions, quality of submission content). Second, we did not ask for feedback from contestants who did not submit high‐quality entries due to limited resources. Youth who were not finalists may have had different experiences and motivations for participating in the crowdsourcing contest. Third, approximately two‐thirds of the total entries were from the younger age group (13−19 years) and the majority were in Russian. This suggests that if one wants to engage diverse groups of AYA, multiple open calls may need to be developed that engage young adults and those who speak only Kazakh. Individuals who speak only Kazakh tend to be predominately located in rural areas of Kazakhstan compared to individuals who are bilingual or speak only Russian, so contestants may have been more urban as well. Finally, each entry was reviewed by four different judges. Inter‐rater agreement was low, suggesting the need for novel approaches to reduce the judging burden and have reliable ratings from a diverse community of judges. Further analyses of study results are ongoing [38] and will be reported in future papers.

## 5 CONCLUSIONS

In summary, citizen science approaches hold great promise for addressing solutions for the increasingly complex health and social challenges facing communities today. Further work is needed to determine for which outcomes citizen science approaches are effective. In the often‐challenging policy environments in EECA health systems, citizen science can be a tool for community change and make interventions more culturally relevant and innovative. Citizen science may also expand citizen knowledge of and trust in science and increase the inclusion of diverse communities. As investigators increasingly use citizen science approaches, it is important that details are shared across studies so that methods can be improved and best practices developed.

## COMPETING INTERESTS

The authors have no competing interests to declare.

## AUTHORS’ CONTRIBUTIONS

AD drafted the paper. All authors reviewed the paper and approved the final manuscript. AD, SLR, JDT, LN, KL, WT, OB, DG, VG, YS, SEL, AT, SP, GM and the JasSpark Study Team were involved in the study and instrument design. OB, DG, VG and GM collected the data. AD, YS, SEL, OB, DG and GM analysed the data. AD and GM obtained study funding.

## FUNDING

This research was supported by the Fogarty International Center and the Eunice Kennedy Shriver National Institute of Child Health and Human Development (R21TW012017). AD is supported by a career development award from the National Institute on Drug Abuse (K01DA044853). JDT is supported by a Mid‐Career Award from the National Institute of Allergy and Infectious Diseases (K24AI143471).

## PERMISSION TO REPRODUCE MATERIAL FROM OTHER SOURCES

All contestants signed a multimedia release form providing permission to share their materials.

## Data Availability

We plan by August 2024 to make JasSpark Study methods, data and results available to the public to the extent that governing data use agreements allow. The data‐sharing plan will comply with the NIH Data Sharing Policy.
